# The Training of Male Nurses

**Published:** 1922-04

**Authors:** John Simonds

**Affiliations:** *Secretary*, National Poor Law Officers' Association Incorporated. Norfolk House, Norfolk Street, Strand, W.C.2.


### The Training of Male Nurses.

*To the Editor of* The Hospital and Health Review.

Sir,—The Executive of this Association has now under consideration the position of male nurses (other than mental nurses), with regard to the General Register of Nurses set up under the Nurses Registration Act, 1919, and I have been directed by my Executive to ask, through the medium of your paper, that any persons interested in this particular question who are in a position to furnish me with information as to any hospitals who train male nurses (other than mental nurses) will be good enough to forward to me particulars of the training schemes.

I should esteem it a favour if you would be good enough to insert this letter in your next issue.

Yours faithfully,
John Simonds,
*Secretary,*
National Poor Law Officers' Association Incorporated.

Norfolk House, Norfolk Street,
Strand, W.C.2.

